# Slow deep breathing modulates cardiac vagal activity but does not affect peripheral glucose metabolism in healthy men

**DOI:** 10.1038/s41598-021-99183-2

**Published:** 2021-10-13

**Authors:** Andreas Vosseler, Dongxing Zhao, Julia Hummel, Ali Gholamrezaei, Sarah Hudak, Konstantinos Kantartzis, Andreas Peter, Andreas L. Birkenfeld, Hans-Ulrich Häring, Robert Wagner, Hubert Preißl, Stephanie Kullmann, Martin Heni

**Affiliations:** 1 Division of Endocrinology, Diabetology and Nephrology, Department of Internal Medicine, Internal Medicine IV, University Hospital Tübingen, Otfried-Müller-Str. 10, 72076 Tübingen, Germany; 2 Institute for Diabetes Research and Metabolic Diseases of the Helmholtz Centre Munich at the University of Tübingen, Tübingen, Germany; 3 German Center for Diabetes Research (DZD), Neuherberg, Germany; 4 Department for Diagnostic Laboratory Medicine, Institute for Clinical Chemistry and Pathobiochemistry, University Hospital Tübingen, Tübingen, Germany; 5 Pain Management Research Institute, Faculty of Medicine and Health, The University of Sydney, Sydney, Australia

**Keywords:** Autonomic nervous system, Medical research, Metabolism

## Abstract

Parasympathetic nervous system innervates peripheral organs including pancreas, hepatic portal system, and gastrointestinal tract. It thereby contributes to the regulation of whole-body glucose metabolism especially in the postprandial state when it promotes secretion of insulin and enhances its action in major target organs. We now aimed to evaluate the effect of parasympathetic modulation on human glucose metabolism. We used slow deep breathing maneuvers to activate the parasympathetic nervous system and tested for effects on metabolism during an oral glucose tolerance test in a randomized, controlled, cross-over trial in 15 healthy young men. We used projections towards the heart as a readout for parasympathetic activity. When analyzing heart rate variability, there was a significant increase of RMSSD (root mean square of successive differences) when participants performed slow deep breathing compared to the control condition, indicating a modulation of parasympathetic activity. However, no statistically significant effects on peripheral glucose metabolism or energy expenditure after the glucose tolerance test were detected. Of note, we detected a significant association between mean heart rate and serum insulin and C-peptide concentrations. While we did not find major effects of slow deep breathing on glucose metabolism, our correlational results suggest a link between the autonomic nervous system and insulin secretion after oral glucose intake. Future studies need to unravel involved mechanisms and develop potential novel treatment approaches for impaired insulin secretion in diabetes.

## Introduction

Insulin resistance of peripheral tissues combined with impaired insulin secretion from pancreatic β-cells are the major pathomechanisms that cause type 2 diabetes^1–4^. One of the main effects of insulin in target tissues is to promote uptake of glucose into the cells. Liver, skeletal muscle and adipose tissue are considered major insulin-sensitive target tissues. By contrast, glucose is absorbed into the brain in an insulin-independent manner. However, insulin receptors are densely expressed in most parts of the central nervous system^5^. Central nervous insulin action modulates the metabolism throughout the body and increases cardiac vagal outputs^6^. Postprandial factors like insulin are perceived by the human brain and induce signals that modulate glucose metabolism via the parasympathetic nervous system^6–8^.

The vagus nerve as major parasympathetic nerve innervates peripheral organs including the pancreas, the hepatic portal system, and most of the gastrointestinal tract. Pancreatic beta cells communicate with vagal sensory neurons in mice^9^. Insulin secretion from the pancreas in pre- and postprandial state is stimulated by vagal efferents^10,11^. Likewise, hepatic insulin sensitivity and insulin clearance is improved upon vagal activation^12^. Accordingly, parasympathetic nervous system modulates glucose metabolism in postprandial state by enhancing insulin sensitivity, insulin secretion and glucose tolerance^6,13,14^.

In a previous study, we investigated the effect of non-invasive transauricular vagus nerve stimulation versus sham stimulation on glucose metabolism during an oral glucose tolerance test^15^. However, the applied methodology was unable to exert major effects on vagus nerve and had therefore no major effects on glucose metabolism. We now aimed to investigate the activation of the parasympathetic nervous system with another approach. Slow deep breathing (SDB) exercise was found to increase parasympathetic activity^16,17^, decrease heart rate ^18^ and alters heart rate variability (HRV) in healthy persons.

We hypothesize that slow deep breathing exercise can modulate peripheral glucose metabolism by alternating vagal responses. To investigate this, we tested effects of slow deep breathing exercise versus normal breathing on insulin sensitivity, insulin secretion, glucose tolerance, resting energy expenditure, and parasympathetic nervous system by analyzing heart rate and heart rate variability.

## Results

### Breathing

We first checked compliance to and efficacy of the breathing instructions. As instructed, the breathing rate significantly decreased during paced-train in the slow deep breathing condition (paced-train vs. pre-train, p_Holm_ = 0.0003), but not in control condition (p_Holm_ = 0.53), and the difference between the two study conditions was significant (p_Holm_ = 0.0002). Moreover, respiration depth significantly increased during paced-train in both slow deep breathing and control conditions (p_Holm_ = 0.0003 and 0.01, respectively). The increase of respiration depth during paced-train was significantly higher in the slow deep breathing compared to the control condition (p_Holm_ = 0.002) (supplementary table 1). The results of mixed model analysis on the effects of slow deep breathing on respiration, heart rate variability and glucose metabolism are presented in Table 1.

**Table 1:** Results of mixed model analysis on the effects of slow deep breathing on respiration, heart rate (variability) and hormones.

	Main effects	Degrees of freedom	*F*	*p*
Breathing rate	Time	28,779	10.99	< 0.0001
	Condition	1,28	16.46	0.0004
	Time-by-condition interaction	28,779	6.71	< 0.0001
Respiration depth	Time	28,779	3.33	< 0.0001
	Condition	1,28	1.97	0.17
	Time-by-condition interaction	28,779	1.54	0.038
Mean heart rate	Time	28,806	10.99	< 0.0001
	Condition	1,29	0.05	0.82
	Time-by-condition interaction	28,806	0.71	0.86
RMSSD	Time	28,806	6.73	< 0.0001
	Condition	1,29	0.10	0.75
	Time-by-condition interaction	28,806	1.42	0.073
Insulin	Time	5,144	198.32	< 0.0001
	Condition	1,29	0.33	0.57
	Time-by-condition interaction	5,144	1.41	0.22
C-peptide	Time	5,144	203.64	< 0.0001
	Condition	1,29	0.02	0.90
	Time-by-condition interaction	5,144	1.18	0.32
Blood glucose	Time	5,144	52.93	< 0.0001
	Condition	1,29	0.00	0.96
	Time-by-condition interaction	5,144	1.10	0.36

### Effects of slow deep breathing on heart rate variability as a proxy for autonomic modulation

**Figure 1:**
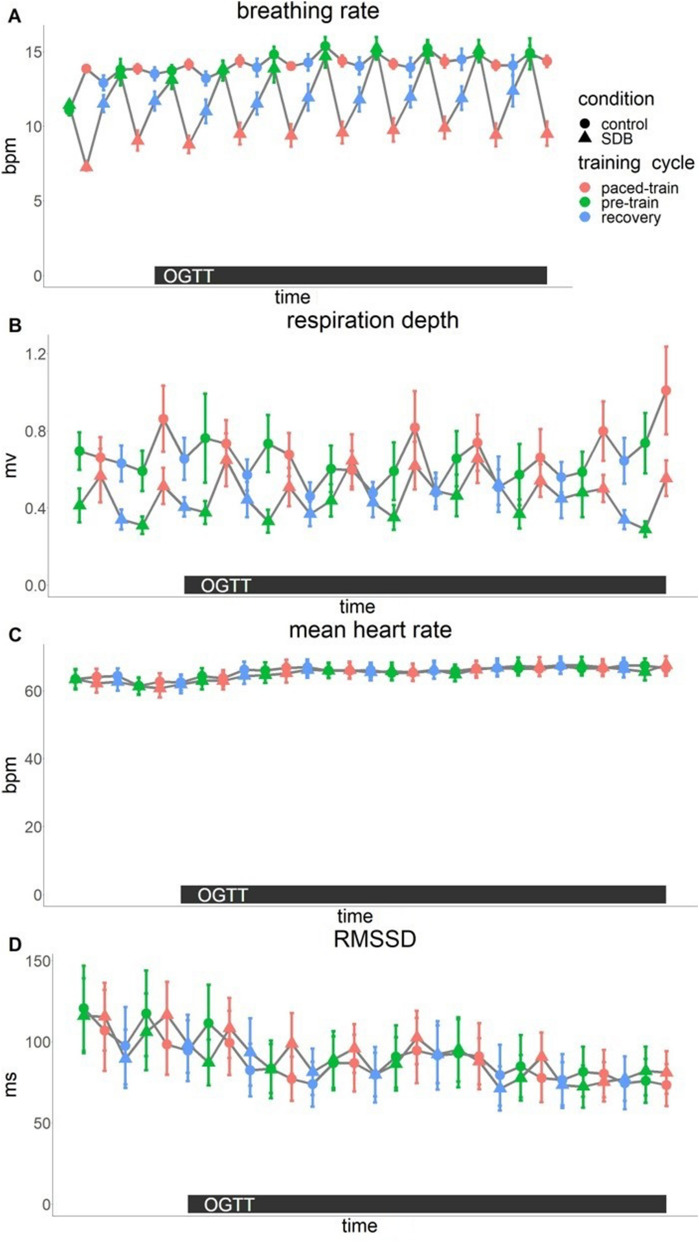
Breathing rate (**A**) and respiration depth (**B**), mean heart rate (**C**) and root mean square of successive differences (RMSSD, **D**). Breathing rate was significantly lower during paced-train compared to pre-train in the slow deep breathing (SDB) condition (p < 0.0005) but not in the control condition (not significant, NS). Respiration depth (averaged by each participant and each condition) was significantly higher during paced-train compared to pre-train in both control and SDB conditions, and the differences between conditions were significant. Mean heart rate (averaged by each participant and each condition) was not significantly different between paced-train and pre-train in either condition. RMSSD increased significantly during paced-train compared to pre-train in SDB condition, but not in control condition, and the differences between the two conditions were significant. OGTT=oral glucose tolerance test; bpm=breaths per minute (A) or beats per minute (C); mv=millivolt; ms=millisecond.

Mean heart rate (HR) did not differ between the conditions (p = 0.82) and there was no significant time-by-condition interaction (p = 0.86) (Fig. 1).

Post hoc contrasts revealed that RMSSD increased in slow deep breathing condition during paced-train cycle compared to pre-train cycle, i.e. when no specific breathing maneuver was performed (p_Holm_ = 0.0003). This was not the case in the control condition (p_Holm_ = 0.16). Moreover, the difference in RMSSD between the conditions was statistically significant (p_Holm_ = 0.0003).

The complete results from the mixed effect models are presented in Table 1. Table 2 displays the results of post hoc contrasts (paced-train cycle vs. pre-train cycles).

**Table 2:** Results of post hoc contrasts (paced-train minus pre-train periods with normal respiration) on the effects of slow deep breathing on respiration and heart rate variability.

	Post hoc contrasts	Degrees of freedom	*t*	*p*_*Holm*_
Breathing rate	SDB	779	− 17.57	0.0003
	Control	779	− 0.63	0.53
	SDB vs. control	779	− 5.36	0.0003
Respiration depth	SDB	799	6.94	0.0003
	Control	799	2.59	0.01
	SDB vs. control	799	3.33	0.002
RMSSD	SDB	806	5.25	0.0003
	Control	806	− 1.74	0.16
	SDB vs. control	806	4.99	0.0003

*RMSSD* root mean square of successive differences.

### Effects of slow deep breathing on whole-body glucose metabolism

Neither fasting glucose, nor post-load glucose were significantly different between the breathing conditions (fasting glucose p = 0.96, post-load glucose p = 0.26). In line, two main determinants of blood glucose levels, insulin secretion and insulin sensitivity were unaffected, as Matsuda insulin sensitivity index (ISI Matsuda) and disposition index (DI) were comparable (ISI Matsuda p = 0.11, DI p = 0.37).

However, statistical models revealed a positive correlation between mean HR and both insulin (p < 0.0001) and C-peptide concentrations (p < 0.0001).

### Effects of slow deep breathing on resting energy expenditure and post-load substrate oxidation

There was no difference in resting energy expenditure between slow deep breathing and normal breathing maneuvers (p = 0.87). Respiratory quotient (RQ) was around 1 during both visits, with no difference between conditions (p = 0.88).

## Discussion

In this study, we investigated the effect of slow deep breathing versus normal breathing during an oral glucose tolerance test on autonomic nervous system activity, on whole-body glucose metabolism, as well as on resting energy expenditure.

Slow deep breathing was performed correctly by the study participants and this appears to have impacted parasympathetic activity as there was an increase of RMSSD upon slow deep breathing. The increase of RMSSD during slow deep breathing indicates a difference between cardiac vagal modulation in the deep breathing versus normal breathing condition.

However, this was not sufficient to introduce major effects on peripheral glucose metabolism or energy expenditure after oral glucose load. The time intervals of deep breathing may have been too short to induce a robust effect on vagal activation and may explain the absence of major metabolic effects. Furthermore, altering cardiac autonomic nervous system by breathing exercise does not necessarily influence its activity at the levels where glucose metabolism can be modulated.

In fact, slow deep breathing was previously shown to modulate autonomic tone when 60 healthy young volunteers practiced deep breathing versus fast breathing for three months^16^. However, this could also be a long-term effect that is not directly relied on slow deep breathing.

In line, Kromenacker et al. showed that slow deep breathing changes heart rate variability predominantly mediated by the parasympathetic nerve and is therefore an effective tool for cardiac vagal activation^28^.

The applied protocol in our study did not change actual heart rate. A prolongation of slow deep breathing (e.g. for 15 min) could eventually be more effective in modulating autonomic tone and may then affect peripheral metabolism. Furthermore, autonomic tone and subsequently peripheral metabolism could also be modulated by pharmacological interventions in future studies.

Another reason for the lack of metabolic effects in our current study may be that slow deep breathing does only modulate specific brain centers that are responsible for the control of autonomic outflow towards the heart. Further autonomic outflows towards metabolic organs are likely under the control of additional brain centers that might not respond to altered breathing patterns^29^.

The tested breathing variation in our current study had no effect on insulinemia. Though, additional analysis revealed a relation of mean heart rate and insulin and C-peptide concentrations regardless of breathing maneuvers or glucose intake. Previous findings affirm the crucial role of the autonomic nervous system for the regulation of insulin and whole-body glucose metabolism^6^. The association of serum C-peptide and insulin levels with heart rate indicates a presumed link between central modulation of insulin secretion and vagal activation of peripheral organs.

Due to the postprandial state (2 h after ingestion of glucose solution) during the measurement of the energy expenditure, RQ indicates glucose as preferential energy source. This preferred energy source was not shifted by the applied breathing protocol.

Our study has some limitations. We currently included only a limited number of participants and were therefore unable to detect smaller effects. Furthermore, only men were included and the breathing protocol might not be strong enough to introduce persistent effects on autonomic tone.

In conclusion, although slow deep breathing has a profound effect on cardiac autonomic activity, it has no major effect on autonomic innervation of other peripheral organs. Insulin secretion, insulin sensitivity and resting energy expenditure were therefore not affected by slow deep breathing in our study. This may be due to an insufficient vagal modulation by the applied slow deep breathing practice or cardiac specificity of vagal responses to this breathing exercise.

The mechanistic basis of the detected association of insulinemia with heart rate and its implication for glucose metabolism warrents to be investigated with further clinical trials. Such results could be the basis to develop novel treatment approaches for impaired insulin secretion in diabetes.

## Methods

In our study, 15 male healthy volunteers between 20 and 55 years were included. Body mass index (BMI) was between 20.5 and 27.3 kg/m^2^, body fat content was measured by bioelectrical impedance testing (BIA 101 by Akern Srl, Florence, Italy) and estimated with Cyprus 2.7 Body Composition Analysis Software (RJL Systems, Michigan, USA). Participants had a body fat content between 6.8 and 20.8 %. Detailed subject characteristics are presented in Table 3.

**Table 3:** Subject characteristics.

n	15
Age (years)	27 (± 8)
BMI (kg/m^2^)	22.7 (± 1.8)
Body fat content (%)	14.0 (± 3.4)
HbA1c (mmol/mol; %)	34,1 (± 2.4); 5.3 (± 0.2)
Waist-to-hip ratio	0.83 (± 0.04)

Values are given as mean ± SD.

With an alpha-level of 0.05, our sample size (n = 15) provided 80% power to detect an effect size f = 0.35 (calculated with Gpower 3.1).

The study protocol was approved by the ethics committee of the medical faculty of the University Tübingen. Written informed consent was obtained from all study volunteers and all research was performed in accordance with relevant guidelines and regulations. The study was pre-registered at clinicaltrials.gov (NCT04150627; 01/11/2019).

**Figure 2:**
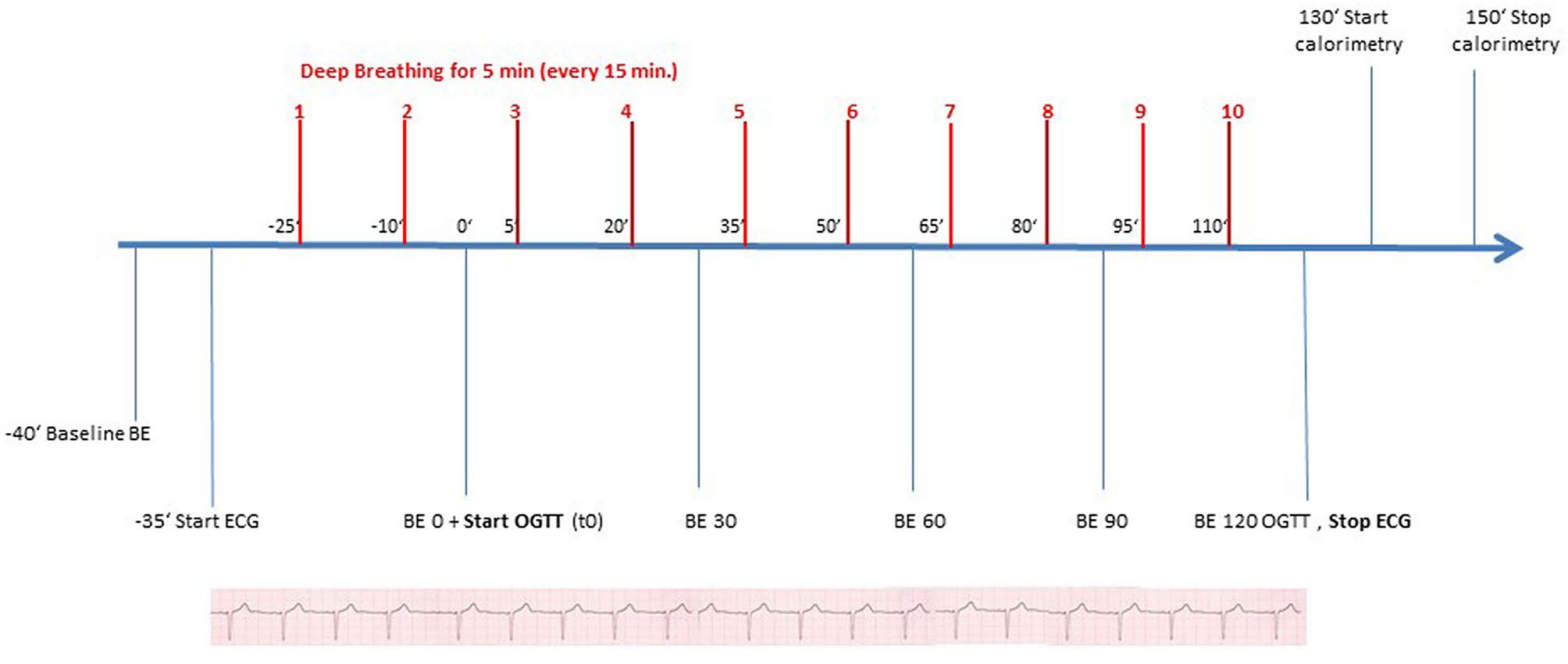
Schematic overview of the experiment. Baseline blood samples were obtained at time point − 40, OGTT was started 40 min after the first blood extraction (BE) and stopped 120 min after ingestion of the glucose solution. ECG recordings were performed 35 min before the start and during the OGTT. Slow deep breathing maneuvers and normal breathing respectively, were performed 25 min before the start of the OGTT for a duration of 5 min with 10 min breaks between each maneuver. Energy expenditure was measured after the end of the OGTT.

**Figure 3:**
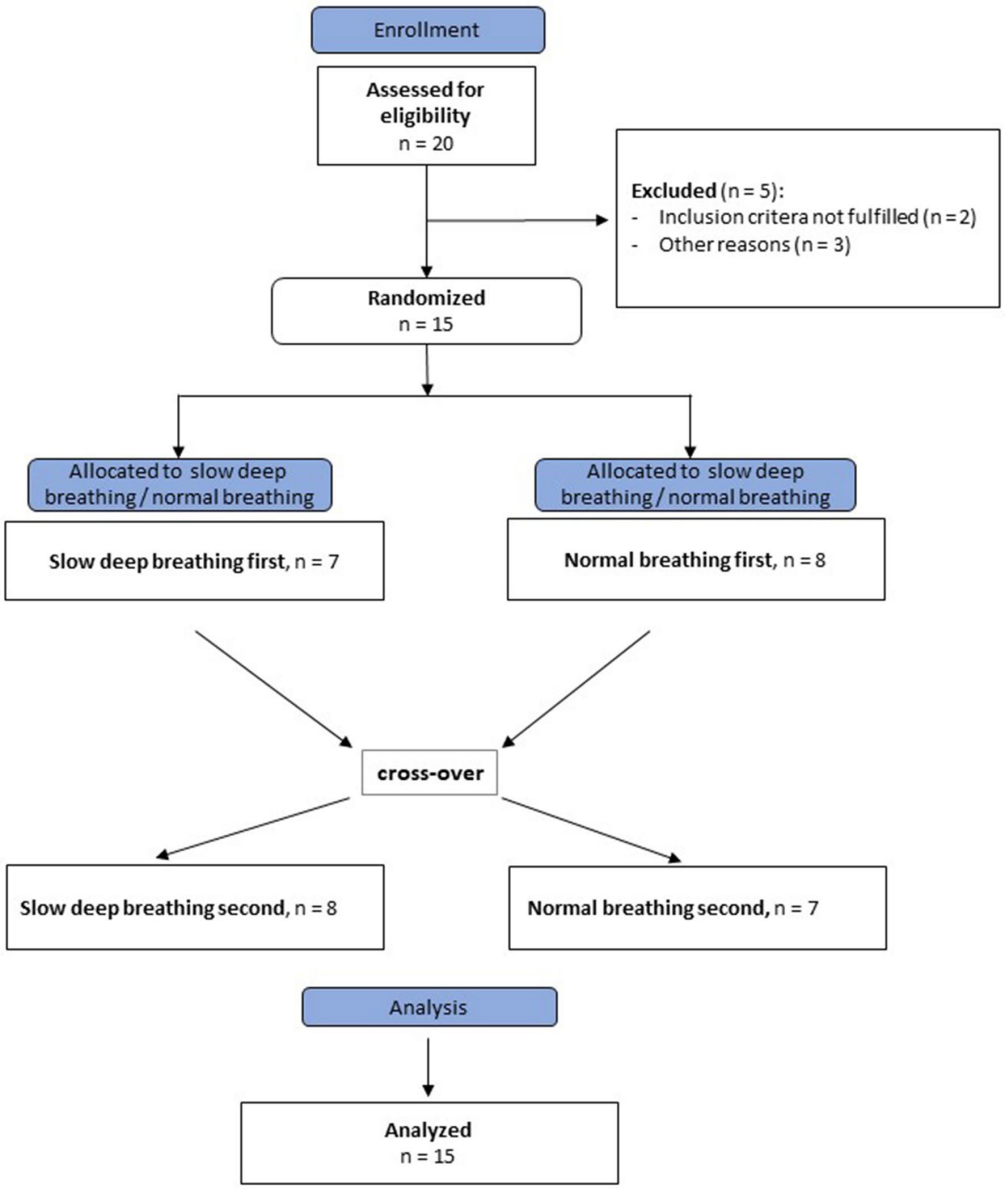
Overview of the course of the study.

A schematic overview of the study is shown in Figs. 2 and 3.

All study participants underwent a 75 g oral glucose tolerance test (OGTT) with blood samples obtained at − 40 min, before glucose ingestion (0 min) and 30, 60, 90 and 120 min after glucose ingestion (Accu-Chek Dextrose OGT, Roche). Glycated hemoglobin (HbA1c) was measured at baseline with Tosoh glycohemoglobin analyzer HLC-723G8 (Tosoh Bioscience, Tokyo, Japan). Glucose, proinsulin and insulin levels, C-peptide, and non-esterified fatty acids (NEFA) were measured at all time points.

Serum pro-insulin, insulin and C-peptide concentrations were determined by an immunoassay with ADVIA Centaur XP Immunoassay System (Siemens Healthineers, Eschborn, Germany). Glucose measurements were done by hexokinase method with ADVIA XPT System (Siemens Healthineers, Eschborn, Germany). All measurements in this study were performed in a routine diagnostic laboratory that is accredited with the German accredited body (DAkkS).

Insulin secretion and insulin sensitivity were calculated from the oral glucose tolerance tests as described previously^19^.

Slow deep breathing or normal breathing was performed twenty-five minutes before and during the entire OGTT in a randomized cross-over design at two different days in the morning.

Volunteers underwent the study in a supine position. Slow deep paced breathing maneuvers vs. control (paced-breathing) were carried out every 15 min for a period of 5 min. Affect 4.0 was used for visually displaying inhalation and exhalation cycles on a laptop with a moving bar for inhalation and exhalation in order to make it easy for the volunteers to follow paced breathing^20^. This also ensured compliance with the breathing rate, inspiration to expiration ratio (1:2) and time intervals. In the Slow deep breathing condition, participants were instructed with 6 breaths per minute during paced breathing. In control condition, participants were instructed with 14 breaths per minute for 5-min followed by a 10-min interval with endogenous breath rate. Hence, a 15-min paced-breathing training cycle (5-min pre-train, 5-min paced-train and 5-min recovery) was repeated until the end of the visit.

Before the start of the experiment, all volunteers were trained in deep breathing and normal breathing maneuvers. Instructions for paced breathing and parameters were based on similar studies showing increased vagal modulation during slow deep breathing^21^.

Breathing cycles were recorded with a respiration belt that was placed around volunteers’ chest (Biopac, Systems Inc., Goleta, CA, USA). Respiration data were collected with Acknowledge Student Lab (Biopac, Systems Inc., Goleta, CA) at a sample rate of 1000 Hz. The depth was measured between the peak and the lowest point before the peak. Collected data were cut into 5-min bins in accordance to the training cycle for analysis in Matlab (Mathworks, Inc. USA). Data segments were preprocessed with a band-pass filter (0.05–1 Hz). Breathing rate (BR) and respiration depth were averaged in each time bin as indicators of volunteers’ respirational activity.

Electrocardiogram (ECG) was recorded to analyze heart rate variability (HRV) as a parameter for sympatho-vagal activation. ECG was continuously recorded throughout the visit with Biopac MP 36 (Biopac Systems, Inc., Goleta, CA), and analyzed in Matlab. Collected data were then cut into 5-min bins in accordance to the training cycle for analysis. ECG was collected with a sampling rate at 1000 Hz. A band-pass filter at 0.5–35 Hz was applied on sampling data. Raw heart rate sampling data were visually inspected and corrected for artifacts in Artiifact^22^, and analyzed in Matlab. Mean heart rate (HR) and root mean square of successive differences (RMSSD) in inter-beat intervals were calculated in the time domain as indicators of the vagal activity. Heart rate variability is mainly modulated by cardiac parasympathetic nerve activity as data of pharmacological blockade of the autonomic nervous system in animals^23,24^ and humans^25,26^ suggest. HRV parameters were determined for each 5-min time period.

Resting energy expenditure was measured after the OGTT and breathing maneuvers. Energy expenditure after deep breathing and normal breathing was calculated by indirect calorimetry measurements with Vyntus CPX (Vyaire Medical, Illinois, USA). Consumption of O_2_ and output of CO_2_ were measured for 20 min. An individual calibration control evaluation (ICcE) was applied, correcting for monitor-specific deviations and eliminating the influence of inherent variability of the device on measurement results^27^.

Statistical analysis was performed using SAS 9.4 (SAS Institute, Cary, NC). p < 0.05 was considered statistically significant, and p < 0.10 is considered a trend. The data are presented as mean ± SE.

Major outcomes were boxcox-transformed. Mixed effect models were performed on respiration and HRV data with main effects of time, condition (slow deep breathing vs. control), and their interaction. Variance–covariance structure providing the best fit was chosen based on the minimum value of Akaike’s Information Criterion (AIC). For breathing rate and respiration depth, planned contrasts were performed to compare between the pre-train and paced-train cycles. For HRV measurements, post hoc contrasts were performed when there was a trend or significant effect of time by condition interaction. The contrasts were performed for the slow deep breathing (SDB) day, the control day, or differences between days (SDB vs. control) respectively, with Bonferroni-Holm correction for multiple testing.

Hormonal results were also analyzed in mixed effect models with main effects of time, condition, and their interaction. Moreover, an additional explorative analysis was performed on hormones with main effect of condition and time, their interaction, and breathing rate, respiration depth, HR and RMSSD (all from the same cycles when the blood samples were collected) as covariates, and reported when covariates were significant.

## Supplementary Information

Supplementary Information.

## Data Availability

The data are not publicly available due to them containing information that could compromise research participant privacy/consent.
