# Supplementary material for: Slow deep breathing modulates cardiac vagal activity but does not affect peripheral glucose metabolism in healthy men
Source: Sci Rep. 2021 Oct 13;11:20306. doi: 10.1038/s41598-021-99183-2 (PMC8514507; doi:10.1038/s41598-021-99183-2)
Supplement: Supplementary file 1 — Supplementary Information. [file 41598_2021_99183_MOESM1_ESM.docx]

**Supplementary table 1. Results of breathing rate, respiration depth and RMSSD in slow deep breathing (SDB) and normal paced breathing conditions, given is mean ± SE. RMSSD: root mean square of successive differences.**

|  |  |  | **breathing rate (per min.)** | | **respiration depth (mV)** | | **mean heart rate (bpm)** | | **RMSSD (ms)** | |
| --- | --- | --- | --- | --- | --- | --- | --- | --- | --- | --- |
| **condition** | **time (min)** | **training cycle** | **Mean** | **Std Error** | **Mean** | **Std Error** | **Mean** | **Std Error** | **Mean** | **Std Error** |
| **SDB**  **-25** | | pre-train | 11.41 | 0.30 | 0.41 | 0.09 | 63.36 | 2.97 | 115.98 | 23.01 |
| **-20** | | paced-train | 7.26 | 0.24 | 0.57 | 0.14 | 62.13 | 2.74 | 115.46 | 20.87 |
| **-15** | | recovery | 11.50 | 0.58 | 0.34 | 0.05 | 62.65 | 2.63 | 89.44 | 17.91 |
| **-10** | | pre-train | 13.46 | 0.74 | 0.31 | 0.05 | 61.29 | 2.51 | 106.01 | 23.68 |
| **-5** | | paced-train | 9.03 | 0.68 | 0.51 | 0.09 | 60.72 | 2.78 | 116.58 | 20.33 |
| **0** | | recovery | 11.67 | 0.64 | 0.40 | 0.05 | 61.83 | 2.58 | 98.67 | 17.88 |
| **5** | | pre-train | 13.10 | 0.62 | 0.38 | 0.06 | 62.90 | 2.55 | 87.09 | 14.11 |
| **10** | | paced-train | 8.77 | 0.58 | 0.65 | 0.14 | 62.91 | 2.54 | 108.42 | 18.46 |
| **15** | | recovery | 10.99 | 0.79 | 0.44 | 0.09 | 64.37 | 2.41 | 93.57 | 20.76 |
| **20** | | pre-train | 13.81 | 0.56 | 0.33 | 0.06 | 64.56 | 2.34 | 82.93 | 17.78 |
| **25** | | paced-train | 9.49 | 0.72 | 0.51 | 0.10 | 65.15 | 2.81 | 98.93 | 18.61 |
| **30** | | recovery | 11.51 | 0.73 | 0.37 | 0.06 | 66.17 | 2.36 | 81.42 | 14.34 |
| **35** | | pre-train | 13.86 | 0.95 | 0.44 | 0.08 | 65.95 | 2.29 | 88.93 | 17.47 |
| **40** | | paced-train | 9.37 | 0.76 | 0.65 | 0.14 | 66.07 | 2.40 | 95.84 | 14.98 |
| **45** | | recovery | 11.91 | 0.89 | 0.43 | 0.08 | 65.41 | 2.39 | 79.79 | 13.44 |
| **50** | | pre-train | 14.67 | 0.76 | 0.35 | 0.06 | 65.73 | 2.49 | 86.23 | 16.34 |
| **55** | | paced-train | 9.57 | 0.74 | 0.62 | 0.12 | 65.42 | 2.56 | 102.57 | 16.48 |
| **60** | | recovery | 11.79 | 0.81 | 0.49 | 0.09 | 66.17 | 2.64 | 92.29 | 17.76 |
| **65** | | pre-train | 15.21 | 0.75 | 0.46 | 0.11 | 64.82 | 2.12 | 95.22 | 19.85 |
| **70** | | paced-train | 9.74 | 0.78 | 0.66 | 0.13 | 66.22 | 2.47 | 87.88 | 14.16 |
| **75** | | recovery | 11.97 | 0.72 | 0.52 | 0.14 | 66.80 | 2.68 | 71.17 | 13.53 |
| **80** | | pre-train | 14.92 | 0.70 | 0.37 | 0.08 | 67.24 | 2.52 | 77.68 | 14.03 |
| **85** | | paced-train | 9.89 | 0.73 | 0.54 | 0.08 | 67.04 | 2.82 | 90.66 | 14.99 |
| **90** | | recovery | 11.88 | 0.79 | 0.45 | 0.10 | 67.46 | 2.53 | 73.32 | 14.12 |
| **95** | | pre-train | 15.11 | 0.66 | 0.48 | 0.13 | 67.43 | 2.53 | 72.36 | 13.00 |
| **100** | | paced-train | 9.40 | 0.77 | 0.50 | 0.07 | 66.87 | 2.39 | 75.24 | 12.19 |
| **105** | | recovery | 12.37 | 1.05 | 0.34 | 0.05 | 66.37 | 2.48 | 77.24 | 13.70 |
| **110** | | pre-train | 14.84 | 1.03 | 0.29 | 0.04 | 65.54 | 2.48 | 81.98 | 14.97 |
| **115** | | paced-train | 9.49 | 0.81 | 0.55 | 0.09 | 67.55 | 2.60 | 80.99 | 13.11 |
| **control**  **-25** | | pre-train | 11.06 | 0.35 | 0.69 | 0.10 | 63.37 | 2.74 | 120.61 | 26.20 |
| **-20** | | paced-train | 13.85 | 0.05 | 0.66 | 0..11 | 64.06 | 2.39 | 106.92 | 24.97 |
| **-15** | | recovery | 12.89 | 0.49 | 0.63 | 0.09 | 64.30 | 2.25 | 97.43 | 23.82 |
| **-10** | | pre-train | 13.77 | 0.71 | 0.59 | 0.10 | 61.42 | 2.46 | 117.46 | 26.28 |
| **-5** | | paced-train | 13.84 | 0.32 | 0.86 | 0.17 | 62.63 | 2.44 | 98.28 | 18.67 |
| **0** | | recovery | 13.50 | 0.44 | 0.65 | 0.11 | 62.43 | 2.33 | 94.44 | 18.80 |
| **5** | | pre-train | 13.72 | 0.35 | 0.76 | 0.23 | 64.25 | 2.37 | 111.37 | 23.58 |
| **10** | | paced-train | 14.12 | 0.31 | 0.73 | 0.12 | 63.69 | 2.40 | 99.23 | 19.75 |
| **15** | | recovery | 13.19 | 0.47 | 0.57 | 0.08 | 66.16 | 2.38 | 82.51 | 16.20 |
| **20** | | pre-train | 13.69 | 0.64 | 0.73 | 0.15 | 66.00 | 2.32 | 83.48 | 15.08 |
| **25** | | paced-train | 14.36 | 0.36 | 0.68 | 0.11 | 66.68 | 2.40 | 77.11 | 13.66 |
| **30** | | recovery | 13.92 | 0.59 | 0.46 | 0.07 | 66.87 | 2.35 | 73.79 | 13.82 |
| **35** | | pre-train | 14.80 | 0.51 | 0.60 | 0.12 | 65.89 | 2.13 | 86.74 | 16.56 |
| **40** | | paced-train | 14.02 | 0.18 | 0.59 | 0.10 | 65.84 | 2.32 | 86.82 | 17.45 |
| **45** | | recovery | 14.26 | 0.55 | 0.48 | 0.06 | 66.06 | 2.22 | 79.65 | 17.10 |
| **50** | | pre-train | 15.36 | 0.60 | 0.59 | 0.15 | 65.24 | 2.19 | 90.76 | 19.17 |
| **55** | | paced-train | 14.36 | 0.37 | 0.82 | 0.19 | 65.33 | 2.38 | 94.46 | 20.05 |
| **60** | | recovery | 14.02 | 0.57 | 0.48 | 0.07 | 65.89 | 2.41 | 91.51 | 21.11 |
| **65** | | pre-train | 14.92 | 0.38 | 0.65 | 0.14 | 65.74 | 1.91 | 92.83 | 20.97 |
| **70** | | paced-train | 14.16 | 0.31 | 0.74 | 0.14 | 66.51 | 2.29 | 91.00 | 20.32 |
| **75** | | recovery | 13.94 | 0.65 | 0.51 | 0.09 | 66.59 | 2.13 | 79.37 | 18.73 |
| **80** | | pre-train | 15.21 | 0.57 | 0.57 | 0.16 | 66.54 | 2.10 | 84.86 | 19.19 |
| **85** | | paced-train | 14.32 | 0.42 | 0.66 | 0.15 | 66.38 | 2.09 | 77.72 | 15.03 |
| **90** | | recovery | 14.47 | 0.72 | 0.56 | 0.08 | 67.16 | 2.15 | 76.51 | 15.83 |
| **95** | | pre-train | 14.79 | 0.52 | 0.59 | 0.10 | 66.55 | 2.14 | 81.49 | 15.46 |
| **100** | | paced-train | 14.07 | 0.32 | 0.80 | 0.15 | 66.48 | 2.21 | 80.32 | 14.60 |
| **105** | | recovery | 14.06 | 0.69 | 0.64 | 0.12 | 67.51 | 2.14 | 74.53 | 16.19 |
| **110** | | pre-train | 14.90 | 0.49 | 0.74 | 0.16 | 67.39 | 2.09 | 75.88 | 13.43 |
| **115** | | paced-train | 14.34 | 0.38 | 1.01 | 0.23 | 66.58 | 2.20 | 73.35 | 12.92 |
